# Flavonoids with inhibitory activity against SARS-CoV-2 3CLpro

**DOI:** 10.1080/14756366.2020.1801672

**Published:** 2020-08-04

**Authors:** Seri Jo, Suwon Kim, Dae Yong Kim, Mi-Sun Kim, Dong Hae Shin

**Affiliations:** a College of Pharmacy, Graduates School of Pharmaceutical Sciences, Ewha W. University, Seoul, Republic of Korea; b N-BIOTEK, Bucheon-Si, Gyeong-gi, Republic of Korea

**Keywords:** SARS-CoV 3CL protease, antiviral, flavonoid, FRET, inhibitory compounds

## Abstract

Coronavirus disease 2019 (COVID-19) has been a pandemic disease of which the termination is not yet predictable. Currently, researches to develop vaccines and treatments is going on globally to cope with this disastrous disease. Main protease (3CLpro) from severe acute respiratory syndrome coronavirus 2 (SARS-CoV-2) is one of the good targets to find antiviral agents before vaccines are available. Some flavonoids are known to inhibit 3CLpro from SARS-CoV which causes SARS. Since their sequence identity is 96%, a similar approach was performed with a flavonoid library. Baicalin, herbacetin, and pectolinarin have been discovered to block the proteolytic activity of SARS-CoV-2 3CLpro. An *in silico* docking study showed that the binding modes of herbacetin and pectolinarin are similar to those obtained from the catalytic domain of SARS-CoV 3CLpro. However, their binding affinities are different due to the usage of whole SARS-CoV-2 3CLpro in this study. Baicalin showed an effective inhibitory activity against SARS-CoV-2 3CLpro and its docking mode is different from those of herbacetin and pectolinarin. This study suggests important scaffolds to design 3CLpro inhibitors to develop antiviral agents or health-foods and dietary supplements to cope with SARS-CoV-2.

## Introduction

Before the nightmares of SARS and MERS has been forgotten, a novel coronavirus causing severe acute respiratory syndrome (SARS-CoV-2) triggered another pneumonia outbreak in Wuhan, China. Now it has posed significant threats to international health and the economy. Coronaviruses (CoVs) are positive single-stranded RNA virus that can infect both animals and humans^1^. Human coronavirus (HCoVs) represents a major group of CoVs associated with various respiratory diseases, from common colds to severe pneumonia and bronchiolitis^2^. Today, HCoV is recognised as one of the fastest-evolving viruses derived from their high element nucleotide replacement rates and recombination^3^. In particular, the virus, which is another characteristic of the past, is making some of the preceding experience obsolete.

A virus genome analysis with a patient with SARS-CoV-2 infection revealed that its nucleotide identities with those of bat SARS-like-CoVZXC21 and human SARS-CoV were ∼89% and ∼82%, respectively^4^^,^^5^. Like the SARS and MERS-CoV genomes, SARS-CoV-2 includes two open reading frames ORF1a and ORF1b, each translated as two viral multi-protein pp1a and pp1ab by host ribosomes. ORF1a encodes two cysteine proteases, a papain-like protease (PLpro) and a 3 C-like protease (3CLpro). PLpro cleaves the first three sites at 181–182, 818–819, and 2763–2764 at the N-terminus and 3CLpro cuts at the remaining 11 sites at the C-terminus, and forming 15 non-structural proteins (nsp)^6^^,^^7^. The crystal structure of 3CLpro from SARS-CoV-2 has been deposited in the Protein Data Bank (6LU7) and revealed the homodimeric form almost similar to that of SARS-CoV. They share common 294 same amino acids out of 306 residues and thus their sequence identity is 96%. Except for two amino acids Ser46 and Val86, none is different in the active site pocket of SARS-CoV-2 3CLpro. The putative cleavage sites of 16 nsp of SARS-CoV-2 predicted by bioinformatics contain QS and QA which are the same amino acid sequences found in 16 non-structural proteins of SARS-CoV. It coincides with the conserved active site together with 96% sequence identity of both 3CLpros. Therefore, in the proteolytic site, both 3CLpros may prefer glutamine at P1 position. P2, P3, and P4 positions may require leucine, basic residues, small hydrophobic residues, respectively^8^. At P1′, P2′, and P3′ positions, small residues are predicted for the two former sites and no strong preference for the last.

In this study, we employed a proteolytic method to probe inhibitory compounds against SARS-CoV-2 3CLpro. A synthetic peptide labelled with an EDANS-DABCYL FRET (Fluorescence resonance energy transfer) pair was used to probe SARS-CoV-2 3CLpro inhibitory compounds. With the FRET pair, an in-house flavonoid library was screened. For a molecular level study, we performed the proteolytic assay with flavonoids followed by an induced-fit docking experiment with top hits. With the results, we investigated a structural and functional relationship of flavonoids endowing binding affinities to SARS-CoV-2 3CLpro. Since the autocleavage process is indispensable with viral propagation, 3CLpro is an attractive drug target for treating COVID-19 patients. The information can be applied to develop synthetic medicine or health-food compounds.

## Materials and methods

### Protein expression and purification

The coding sequence of SARS-CoV-2 3 C-like proteinase (NCBI Ref. seq. YP_009725301.1) was synthesised chemically by Bioneer (Daejeon, Korea) and cloned into a bacteriophage T7-based expression vector. The plasmid DNA was transformed into *E. coli* BL21 (DE3) for protein expression. *E. coli* BL21 (DE3) cells were grown on Luria–Bertani (LB) agar plates containing 150 *μ*g ml^−1^ ampicillin. Several colonies were picked and grown in capped test tubes with 10 ml LB broth containing 150 *μ*g ml^−1^ ampicillin. A cell stock composed of 0.85 ml culture and 0.15 ml glycerol was prepared and frozen at 193 K for use in a large culture. The frozen cell stock was grown in 5 ml LB medium and diluted into 1000 ml fresh LB medium. The culture was incubated at 310 K with shaking until an OD_600_ of 0.6–0.8 was reached. At this point, the expression of SARS-CoV-2 3CLpro was induced using isopropyl-β-d-1-thiogalactopyranoside (IPTG) at a final concentration of 1 mM. The culture was further grown at 310 K for 3 h in a shaking incubator. Cells were harvested by centrifugation at 7650 *g* (6500 rev min^−1^) for 10 min in a high-speed refrigerated centrifuge at 277 K. The cultured cell paste was resuspended in 25 ml of a buffer consisting of 50 mM Tris–HCl pH 8.0, 100 mM NaCl, 10 mM imidazole, 1 mM phenylmethylsulfonyl fluoride (PMSF), 10 *μ*g ml^−1^ DNase I. The cell suspension was disrupted using an ultrasonic cell disruptor (Digital Sonifier 450, Branson, USA). Cell debris was pelleted by centrifugation at 24,900 *g* (15,000 rev min^−1^) for 30 min in a high-speed refrigerated ultra-centrifuge at 277 K.

The protein was purified by affinity chromatography using a 5 ml Hi-Trap Q His column (GE Healthcare, Piscataway, New Jersey, USA). The column was equilibrated with a buffer consisting of 20 mM Bis-Tris pH 7.5. The column was eluted using a linear NaCl gradient to 1.0 M NaCl and the protein was eluted at 0.18 M NaCl.

The purified protein was buffer exchanged into 20 mM Bis-Tris pH 7.5 using Vivaspin 20 MWCO 10 kDa (GE Healthcare), a centrifugal device. SDS–PAGE showed one band around 34 kDa (33796.64 Da), corresponding to the molecular weight of SARS-CoV-2 3CLpro.

### FRET protease assays with SARS-CoV-2 3CLpro

The custom-synthesised fluorogenic substrate, DABCYL-KTSAVLQSGFRKME-EDANS (ANYGEN, Gwangju, Korea), was used as a substrate for the proteolytic assay using the SARS-CoV-2 3CLpro^9^. This substrate contains the nsp4/nsp5 cleavage sequence, GVLQ↓SG^10^, and works as a generic peptide substrate for many coronaviruses including the SARS-CoV-2 3CLpro. The peptide was dissolved in distilled water and incubated with each protease. A SpectraMax i3x Multi-mode microplate reader (Molecular Devices) was used to measure spectral-based fluorescence. The proteolytic activity was determined at 310 K by following the increase in fluorescence (λ_excitation_ = 340 nm, λ_emission_ = 490 nm, bandwidths = 9, 15 nm, respectively) of EDANS upon peptide hydrolysis as a function of time. Assays were conducted in black, 96-well plates (Nunc) in 300 *μ*l assay buffers containing protease and substrate as follow; For the SARS-CoV-2 3CLpro assay, 2.04 *μ*l of 0.294 mM protease containing 20 mM Tris pH 7.5 was incubated with 7.5 *μ*l of 0.1 mM substrate at 310 K for 2 h 30 min before measuring Relative Fluorescence Units (RFU). Before the assay, the emission spectra of antiviral agents and some of their adjuvants were surveyed after illuminating at 340 nm to avoid the overlapping with the emission spectrum of EDANS. Every compound was suitable to be tested. The final concentration of the protease, peptide, and chemical used at the assay was 2 *µ*M, 2.5 µM and 80 *µ*M each. At first, SARS-CoV-2 3CLpro and chemical were mixed and preincubated at room temperature for 1 h. The reaction was initiated by the addition of the substrate and each well was incubated at 310 K for 2 h 30 min. After that, we measured the fluorescence of the mixture on the black 96-well plate using the endpoint mode of SpectraMax i3x where the excitation wavelength was fixed to 340 nm and the emission wavelength was set to 490 nm using 9, 15 nm bandwidth, respectively. All reactions were carried out in triplicate. Among the first seventy flavonoids (Supplementary Table 1), one of them was picked up to further assay at a concentration range of 4 *µ*M ∼ 240 *µ*M. The IC_50_ value which is the value causing 50% inhibition of SARS-CoV-2 3CLpro was calculated by nonlinear regression analysis using GraphPad Prism 7.03 (GraphPad Software, San Diego, CA, USA).

### FRET protease assays with SARS-CoV-2 3CLpro in the presence of triton X-100

The proteolytic assay using SARS-CoV-2 3CLpro in the presence of Triton X-100 has been performed to differentiate the artificial inhibitory activity of chemicals through non-specific binding with proteases by forming aggregate or complexation. The concentration used in this study was 0.01%.

### Absorption spectroscopic studies based on tryptophans of SARS-CoV-2 3CLpro

To confirm the feasibility of the assay method independently, the fluorescence spectra from tryptophans of SARS-CoV-2 3CLpro with candidate inhibitors were investigated^11^. The fluorescence measurements were recorded with a SpectraMax i3x Multi-mode microplate reader (Molecular Devices) at excitation and emission wavelengths of 295 nm and 320–500 nm, respectively. The optimal excitation and emission wavelengths were determined by SoftMax Pro. Three tryptophans of SARS-CoV-2 3CLpro showed a fluorescence emission with a peak at 340 nm after the excitation at the wavelength of 295 nm. In contrast, the flavonoids were almost non-fluorescent under the same experiment condition. Each 80 *µ*M chemical was incubated with 2 *µ*M SARS-CoV-2 3CLpro for 1 h and the fluorescence intensity of the mixture was measured.

### Ligand preparation, target preparation, and induced-fit docking

All the docking and scoring calculations were performed using the Schrödinger software suite (Maestro, version 11.8.012). The compounds were extracted from the PubChem database in the SDF format and were combined in one file. The file was then imported into Maestro and prepared for docking using LigPrep. The atomic coordinates of the crystal structure of SARS-CoV-2 3CLpro (6LU7) were retrieved from the Protein Data Bank and prepared by removing all solvent and adding hydrogens and minimal minimisation in the presence of bound ligand using Protein Preparation Wizard. Ioniser was used to generate an ionised state of all compounds at the target pH 7.0 ± 2.0. This prepared low-energy conformers of the ligand were taken as the input for an induced-fit docking. The induced-fit docking protocol^12^ was run from the graphical user interface accessible within Maestro 11.8.012. Receptor sampling and refinement were performed on residues within 5.0 Å of each ligand for each of the ligand–protein complexes. With Prime^13^, a side‐chain sampling and prediction module, as well as the backbone of the target protein, were energy minimised. A total of induced‐fit receptor conformations were generated for each of the ligands. Re‐docking was performed with the test ligands into their respective structures that are within 30.0 kcal/mol of their lowest energy structure. Finally, the ligand poses were scored using a combination of Prime and GlideScore scoring functions^14^.

## Results

The cell yield harvested for purification of SARS-CoV-2 3CLpro was 2.9 g per 2000 ml of *E. coli* culture. The amount of purified protein synthesised was 29.85 mg. For storage and assay, the protein solution was concentrated to 99.5 mg ml^−1^. The concentrate was diluted to 2 *µ*M when the inhibitory assay was carried out.

**Figure 1.**
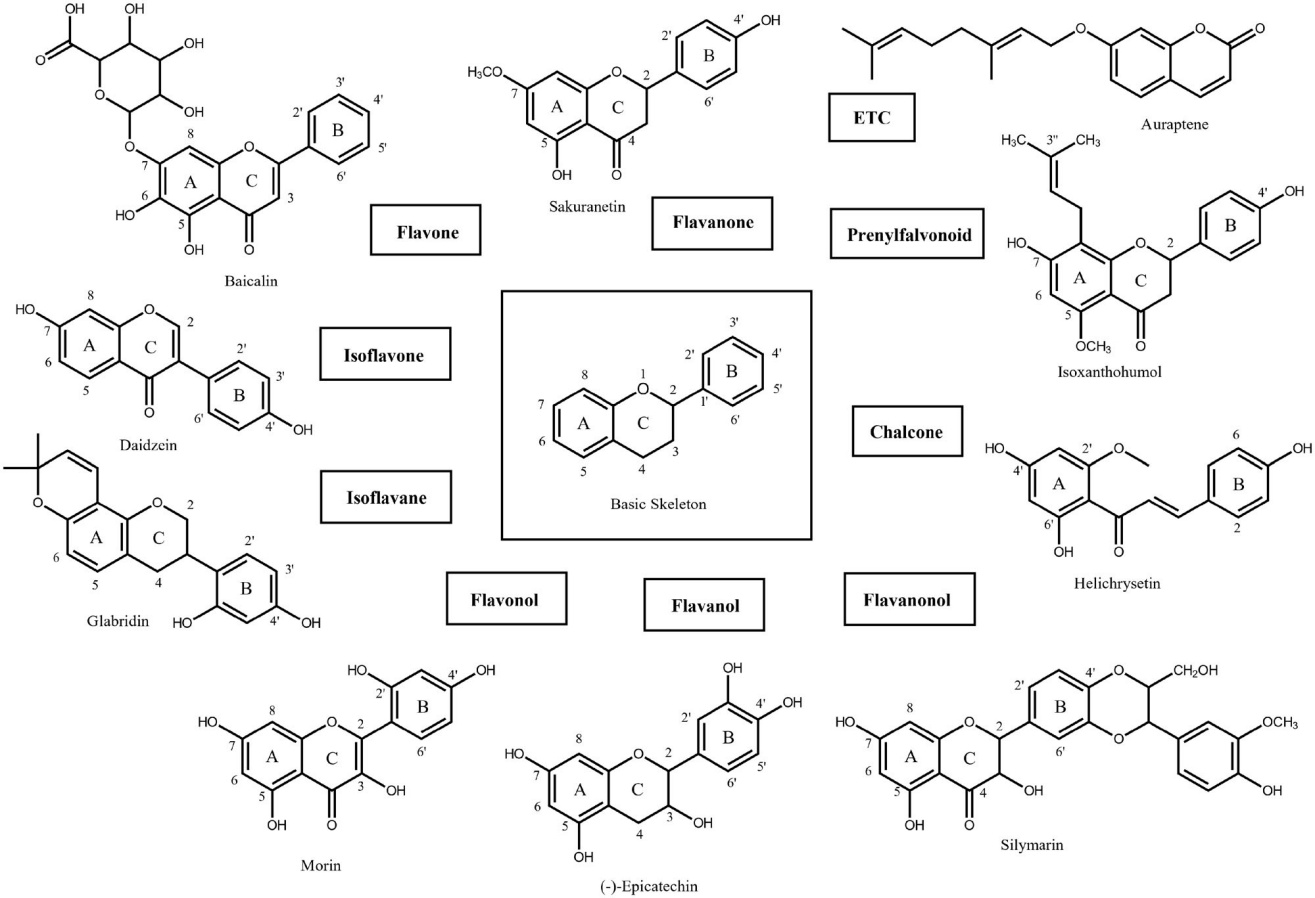
The basic skeleton structures of flavonoids and their scaffolds. Basic representative structures of the most common flavonoids classified in this study were drawn with rings and numbered positions.

**Figure 2.**
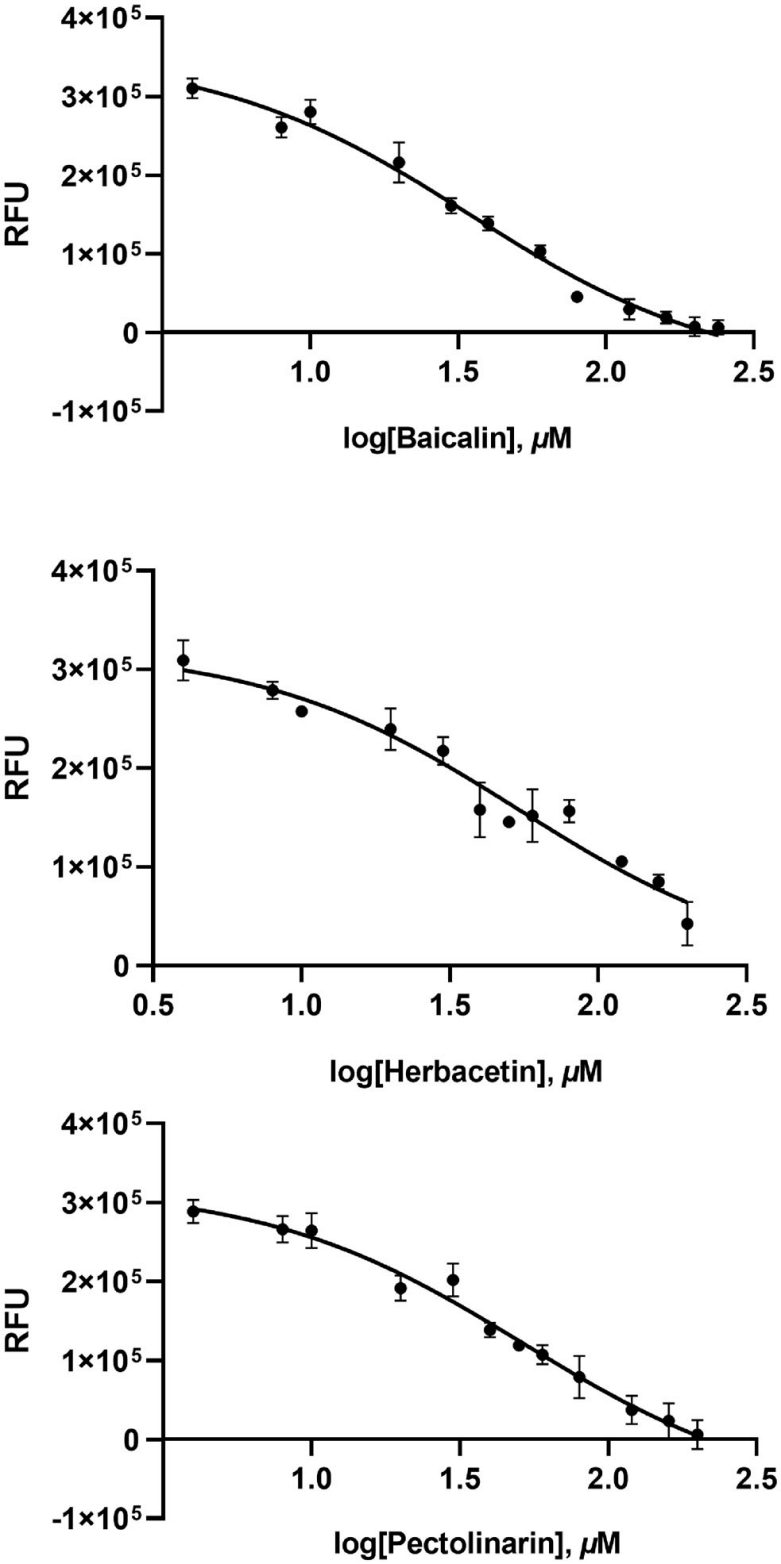
Results from the FRET method. Each data point represents the effect of each inhibitory compound against SARS-CoV-2 3CLpro compared to the control. The RFU are plotted against the log-concentration of inhibitory compounds. Each dot is expressed as the mean ± standard error of the mean (*n* = 3). RFU: relative fluorescence units.

A flavonoid library consisting of 10 different scaffolds was also built (Figure 1). It contains 5 isoflavones, 1 isoflavane, 18 flavones, 16 flavonols, 7 flavanols, 7 flavanones, 4 flavanonol, 1 prenylflavonoid, 9 chalcones, and 2 unclassified flavonoids (Supplementary Table 1). We applied the library to assay SARS-CoV-2 3CLpro. Using 70 flavonoids, an inhibitory effect of each compound at 80 *µ*M was tested. Five flavones; orientin (8-β-d-glucopyranosyl-3′,4′,5,7-tetrahydroxyflavone), baicalin (7-d-glucuronic acid-5,6-dihydroxyflavone), pectolinarin (5,7-dihydroxy 4′,6-dimethoxyflavone 7-rutinoside), homoplantaginin (6-methoxyapigenin 7-*O*-glucoside), rhoifolin (apigenin-7-*O*-rhamnoglucoside) and two flavonols; herbacetin (3,4′,5,7,8-pentahydroxyflavone), rutin (3,3′,4′,5,7-Pentahydroxyflavone-3-rutinoside) were found to have inhibitory activity. Among them, baicalin, herbacetin, and pectolinarin were found to have prominent inhibitory activity. The binding affinity data were plotted as log inhibitor concentration *versus* percent fluorescence inhibition (Figure 2). The compound showed the severely reduced fluorescent intensity and thus represented their SARS-CoV-2 3CLpro inhibitory activity. The IC_50_ values were calculated from the dose-dependent inhibitory curves of baicalin, herbacetin and pectolinarin. The measured values were 34.71 *µ*M, 53.90 *µ*M and 51.64 *µ*M, respectively. Since flavonoids are known to be aggregated through complexity and thus non-specifically inhibit various proteases, the assay in the presence of Triton X-100 was also performed^15^. Before the examination, we tested the effects of Triton X-100 on SARS-CoV-2 3CLpro. There was no difference in catalyst activity with or without 0.01% Triton X-100. Therefore, the assay was performed at a concentration of 0.01% Triton X-100.

**Figure 3.**
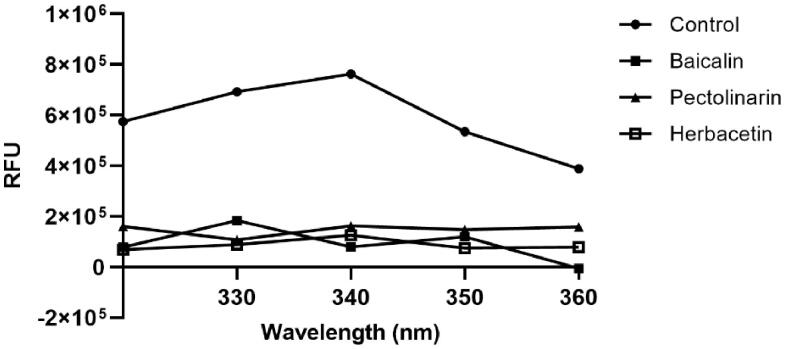
Fluorescence quenching spectra of SARS-CoV-2 3CLpro. A solution containing 2 *μ*M SARS-CoV 3CLpro showed a strong fluorescence emission with a peak at 340 nm at the excitation wavelength of 295 nm. After adding 80 *μ*M each inhibitory compound such as baicalin, herbacetin, and pectolinarin, fluorescence quenching spectra were obtained.

To independently confirm the inhibitory activity of flavonoids, a general tryptophan based assay method was employed. Tryptophan was well known to emit its fluorescence. Thus, if the tryptophan is properly located in the protein, the change in fluorescence intensity can reflect the binding state of the chemicals and can be used to determine the interaction between the protein and the chemical. The SARS-CoV-2 3CLpro contains three tryptophan residues. Therefore, its fluorescence change can reflect the environmental variation of the protein. The SARS-CoV-2 3CLpro used in this study displays a fluorescence peak at 340 nm after the tryptophan excitation wavelength of 295 nm. We monitored the change of the fluorescence intensities depending on the presence or absence of all flavonoids. Since each compound in the flavonoid library was almost non-fluorescent under experimental conditions, the change in fluorescence intensity reflects the interaction between the protein and chemical. The decreased emission intensity confirmed the complex formation between the SARS-CoV-2 3CLpro and the inhibitory compound (Figure 3).

## Discussion

Flavonoids constitute a large class of food constituents influencing a positive impact on health. Many flavonoids occur as large molecules called tannins. These flavonoids form polymers through various carbon–carbon and ether linkages. Flavonoids are important kinds of natural products widely found in fruits and vegetables. A wide range of beneficial roles with antioxidants, anti-inflammatory, anti-mutagens, and anti-cancer-causing properties have been reported against various diseases such as Parkinson’s disease, Alzheimer’s disease, and cancer^16–20^. Intriguingly, some flavonoids possess antiviral activity and some of their direct proteolytic inhibitory activity against SARS-CoV 3CLpro has been published^21^.

The recent pandemic caused by SARS-CoV-2 is going on severely and thus patients with COVID-19 are exponentially increasing. Therefore, the development of vaccines and antiviral agents against SARS-CoV-2 is urgent. Currently, some compounds designed for other RNA viruses such as favipiravir, lopinavir, and tenofovir have been used in clinical trials in patients with COVID-19 (http://clinicaltrials.gov). Therefore, designing chemicals targeting directly against specific enzymes of SARS-CoV-2 is very important. As mentioned above 3CLpro of SARS-CoV-2 is a good target to design inhibitory chemicals since some flavonoids inhibit the proteolytic activity of SARS-CoV 3CLpro^21^. The 96% sequence identity between them together with their conserved active site suggests that similar flavonoids may work for SARS-CoV-2 3CLpro.

The full sequence of SARS-CoV-2 3CLpro was purified and assayed against the in-house flavonoid library composed of various flavonoid derivatives with ten different scaffolds. The promising compounds include five flavones orientin, baicalin, pectolinarin, homoplantaginin and rhoifolin and two flavonols; herbacetin, rutin. Among them, baicalin, herbacetin and pectolinarin revealed the prominent inhibitory activity against SARS-CoV-2 3CLpro. The measured IC50 values were 34.71, 53.90 and 51.64 *µ*M, respectively.

An *in silico* docking study for baicalin, herbacetin and pectolinarin was performed to deduce their binding mode and binding affinity. The glide scores of three compounds were −8.776, −8.738 and −10.969, respectively. The binding modes of herbacetin and pectolinarin were similar to those obtained from the docking study of the catalytic domain of SARS-CoV 3CLpro^21^. In the case of herbacetin, the phenyl moiety occupies the S1 site of SARS-CoV-2 with the aid of Glu166 and the chromen-4-one scaffold locates in the S2 site with hydrogen bonds with His41 and Gln189. The binding mode of pectolinarin showed that the l-mannopyranosyl β-d-glucopyranoside moiety occupies the S1 and S2 sites and the chromen-4-one moiety locates in the S2 and S3′ site similar to the binding mode observed in SARS-CoV 3CLpro.

**Figure 4.**
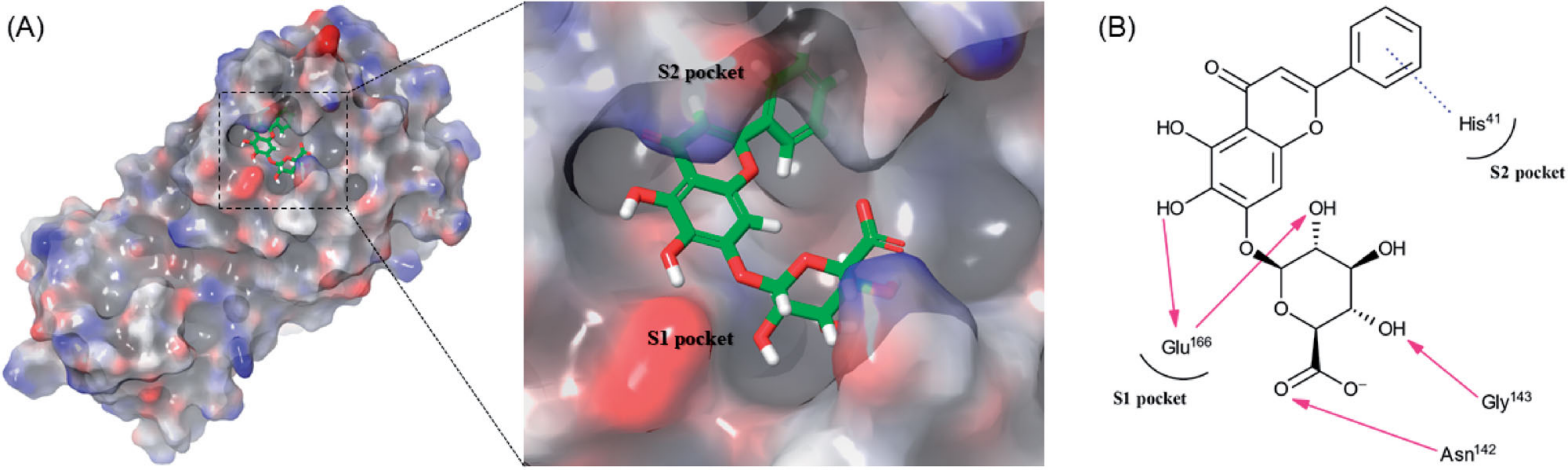
(A) Schematic representation of baicalin docked on the catalytic cavity of SARS-CoV-2 3CLpro. The electrostatic surface potential of SARS-CoV-2 3CLpro docked with baicalin was depicted (red, negative; blue, positive; white, uncharged). The enlarged view represents baicalin in the active site pocket with a semi-transparent view of the molecular surface of SARS-CoV-2 3CLpro. (B) A 2D schematic representation of the interactions of baicalin with the side chains of SARS-CoV-2 3CLpro. The pink arrows represented hydrogen bonds and the blue dot line for π–π stacking.

The binding mode of baicalin is described in Figure 4. Due to the presence of the glucuronate moiety, its binding mode is more similar to that of pectolinarin than herbacetin. The important hydrogen bonds with Glu166 are formed by the 6-hydroxyl group attached to the chromen-4-one moiety (2.778 Å) and the 5-hydroxyl group attached to the glucuronate moiety (3.331 Å). As a result, more than half of the compound was docked in the S1 site. The two hydrogen bonds of the 3-hydroxyl and carboxylic acid groups from the glucuronate moiety formed with Gly143 (2.962 Å) and Asn142 (2.883 Å), respectively, stabilise the overall interaction. The π–π stacking between His41 and the phenyl moiety is first observed in baicalin.

In the previous results of SARS-CoV 3CLpro^21^, only the three effect flavonoids (herbacetin, pectolinarin, and rhoifolin) were mentioned. However, other four flavonoids (orientin, baicalin, homoplantaginin, and rutin) were also detected though their efficiency was low (data not shown). It is actually expected due to the 96% sequence identity of the two 3CLpro. Interestingly, the affinity of rhoifolin became weaken but the affinity of baicalin became stronger with SARS-CoV-2 3CLpro. The efficiency of two flavonoids (herbacetin and pectolinarin) was still promising. Considering almost the same active site composition of both 3CLpros, the different binding affinity seemed to be a bit strange. However, this difference could be explained by the two factors. At first, the difference may be originated from the different constructs used in both cases. In this study, we used the whole SARS-CoV-2 3CLpro but the catalytic domain of SARS-CoV 3CLpro (amino acids Met1-Thr196) had been used in the previous study. In the crystal structures of both 3CLpros, there is a dimerisation domain influencing proteolytic activity. That domain was not in the previous SARS-CoV 3CLpro study. Nevertheless, a long incubation result showed that the proteolytic function of the SARS-CoV 3CLpro is still present without the dimerisation domain. Second, there are some different residues comprising active site residues. Ser46 and Val86 in SARS-CoV-2 3CLpro are replaced by Ala46 and Leu86 in SARS-CoV 3CLpro, respectively. Though their substitution seems to be minor in amino acid properties, the different residues may clearly contribute different affinities.

The assay and docking result indicates important conclusions. At first, flavonoids have a wide range of binding affinity to SARS-CoV-2 3CLpro as observed in SARS-CoV 3CLpro^21^. Second, the presence of the monocarbohydrate derivative, glucuronate, observed in baicalin severely influences to its mode compared with herbacetin and pectolinarin. The unique binding mode of baicalin can be applied to newly design inhibitory compounds against SARS-CoV-2 3CLpro with the different scaffold. A further study is going on to make derivatives that lead to better inhibitory compounds and develop health-foods and dietary supplements to cope with SARS-CoV-2 based on this study.

## Supplementary Material

Supplemental Material
