# Supplementary material for: Flavonoids with inhibitory activity against SARS-CoV-2 3CLpro
Source: J Enzyme Inhib Med Chem. 2020 Aug 4;35(1):1539–44. doi: 10.1080/14756366.2020.1801672 (PMC7470085; doi:10.1080/14756366.2020.1801672)
Supplement: Supplemental Material [file IENZ_A_1801672_SM6712.pdf]

Supplementary Table 1. A flavonoid library

No	Name of compound	No	Name of compound
1-1	Daidzein	4-12	Myricitrin
1-2	Genistein	4-13	Kaempferide
1-3	Genistin	4-14	Galangin
1-4	Ipriflavone	4-15	Chrysin
1-5	Puerarin	4-16	Wogonin
2-1	Glabridin	5-1	(−)-Gallocatechin
3-1	Baicalein	5-2	(±)-Epigallocatechin gallate
3-2	Diosmin	5-3	(−)-Epicatechin
3-3	Diosmetin	5-4	(±)-Catechin
3-4	Skullcapflavone II	5-5	(−)-Gallocatechin gallate
3-5	beta-Naphthoflavone	5-6	(−) Catechin gallate
3-6	Orientin	5-7	(+)-Catechin hydrate
3-7	Acacetin	6-1	Hesperidin
3-8	Baicalin	6-2	Naringenin
3-9	Rhoifolin	6-3	Sakuranetin
3-10	Hispidulin	6-4	Naringin
3-11	Sinensetin	6-5	Poncirin
3-12	Oroxin B	6-6	Bavachin
3-13	Pectolinarin	6-7	Flavanone
3-14	Cirsiliol	7-1	(±)-Taxifolin hydrate
3-15	Homoplantaginin	7-2	Silibinin
3-16	Amentoflavone	7-3	Astilbin
3-17	Luteolin	7-4	Silymarin
3-18	Apigenin	8-1	Isoxanthohumol
4-1	Herbacetin	9-1	Isobavachalcone
4-2	Kaempferol	9-2	2,2′,4′-Trihydroxychalcone
4-3	Morin	9-3	Dienestrol
4-4	Myricetin	9-4	Sofalcone
4-5	Fisetin	9-5	Rhodamine 6G
4-6	Quercitrin	9-6	FCLA Free Acid
4-7	Quercetin	9-7	Helichrysetin

4-8	Quercetin 3-β-D-glucoside	9-8	Cardamonin
4-9	Kaempferol 7-O-β-D-glucopyranoside	9-9	Neohesperidin dihydrochalcone
4-10	Rutin	10-1	Mangiferin
4-11	Icaritin	10-2	Auraptene

*Isoflavone;1-1~5, Isoflavane;2-1, Flavone;3-1~18, Flavonol;4-1~16, Flavanol;5-1~7, Flavanone;6-1~7, Flavanonl;7-1~4, Prenylflavonoid;8-1, Chalcone;9-1~9, unclassified;10-1~2
